# Assessment of mortality and performance status in critically ill cancer patients: A retrospective cohort study

**DOI:** 10.1371/journal.pone.0252771

**Published:** 2021-06-11

**Authors:** Esther N. van der Zee, Lianne M. Noordhuis, Jelle L. Epker, Nikki van Leeuwen, Bas P. L. Wijnhoven, Dominique D. Benoit, Jan Bakker, Erwin J. O. Kompanje

**Affiliations:** 1 Department of Intensive Care, Erasmus University Medical Center, Rotterdam, The Netherlands; 2 Department of Public Health, Center for Medical Decision Making, Erasmus University Medical Center, Rotterdam, The Netherlands; 3 Department of Surgery, Erasmus University Medical Center, Rotterdam, The Netherlands; 4 Department of Intensive Care, Ghent University Hospital, Ghent, Belgium; 5 Department of Pulmonology and Critical Care, New York University NYU Langone Medical Center, New York, NY, United States of America; 6 Department of Pulmonology and Critical Care Columbia University Irvine Medical Center, New York, NY, United States of America; 7 Department of Intensive Care, Pontificia Universidad Católica de Chile, Santiago, Chile; University of South Carolina College of Pharmacy, UNITED STATES

## Abstract

**Introduction:**

Given clinicians’ frequent concerns about unfavourable outcomes, Intensive Care Unit (ICU) triage decisions in acutely ill cancer patients can be difficult, as clinicians may have doubts about the appropriateness of an ICU admission. To aid to this decision making, we studied the survival and performance status of cancer patients 2 years following an unplanned ICU admission.

**Materials and methods:**

This was a retrospective cohort study in a large tertiary referral university hospital in the Netherlands. We categorized all adult patients with an unplanned ICU admission in 2017 into two groups: patients with or without an active malignancy. Descriptive statistics, Pearson’s Chi-square tests and the Mann-Whitney U tests were used to evaluate the primary objective 2-year mortality and performance status. A good performance status was defined as ECOG performance status 0 (fully active) or 1 (restricted in physically strenuous activity but ambulatory and able to carry out light work). A multivariable binary logistic regression analysis was used to identify factors associated with 2-year mortality within cancer patients.

**Results:**

Of the 1046 unplanned ICU admissions, 125 (12%) patients had cancer. The 2-year mortality in patients with cancer was significantly higher than in patients without cancer (72% and 42.5%, P <0.001). The median performance status at 2 years in cancer patients was 1 (IQR 0–2). Only an ECOG performance status of 2 (OR 8.94; 95% CI 1.21–65.89) was independently associated with 2-year mortality.

**Conclusions:**

In our study, the majority of the survivors have a good performance status 2 years after ICU admission. However, at that point, three-quarter of these cancer patients had died, and mortality in cancer patients was significantly higher than in patients without cancer. ICU admission decisions in acutely ill cancer patients should be based on performance status, severity of illness and long-term prognosis, and this should be communicated in the shared decision making. An ICU admission decision should not solely be based on the presence of a malignancy.

## Introduction

Over the past two decades, the number of cancer patients requiring intensive care treatment has increased [1]. In 2009, a large European multicentre study showed that 15% of the patients admitted to the Intensive Care Unit (ICU) have cancer [2]. Historically, during the 1980’s and 1990’s, these patients were commonly considered ineligible for intensive care treatment given their presumed unfavourable outcome [3,4]. However, ICU and hospital mortality of these patients has decreased significantly [1,4–6]. Nowadays, ICU patients with a solid malignancy have similar mortality when compared to ICU patients without a malignancy [7]. Although the reported mortality of ICU patients with a hematological malignancy varies [8–10], a decrease in mortality over the years has been described [5,11].

Despite the encouraging trend in survival rates of cancer patients admitted to the ICU, the decision to admit these patients to the ICU remains challenging, especially in the acute setting [12]. In an area with rapid evolution of diagnostics and new innovative cancer treatments, reliable literature about mortality and morbidity of cancer patients admitted to the ICU unplanned is essential, in order to manage outcome expectations of health care providers, patients and relatives.

Complications of cancer treatment, such as cardiomyopathy or kidney failure, can become clinically evident even decades after completion of therapy [13–17]. These complications could influence the outcome of former cancer patients admitted to the ICU. Mortality and performance status of former cancer patients in complete remission (CR) requiring an unplanned ICU admission has not been extensively studied, except for stem cell transplant patients with hematologic malignancies.

The aim of this study was to assess the mortality and performance status of critically ill cancer patients 2 years after an unplanned ICU admission. Second, we aimed to compare mortality of these acutely admitted patients to mortality of patients without cancer. Further, we aimed to identify factors associated with 2-year mortality in cancer patients. Finally, we assessed mortality of former cancer patients in complete remission at time of ICU admission.

## Materials and methods

### Study design and setting

We conducted a retrospective cohort study in a large tertiary referral university hospital ICU in the Netherlands. First, we identified all adult patients with an unplanned admission to the ICU in 2017, using the Electronic Health Record (EHR). Subsequently, we categorized the patients into two groups: 1) patients with an active malignancy (study population), 2) patients without active cancer. Only for the analyses evaluating outcomes of patients in CR, a third group was created, in which former cancer patients were included.

### Participants

All patients with an unplanned ICU admission and active cancer were included in group 1, the study population. All patients without cancer were included in group 2. Only for the analyses regarding patients in CR, the latter group was divided in patients without cancer and former cancer patients.

We excluded patients with a diagnosis of a non-melanoma skin malignancy (squamous-cell skin cancer or basal-cell carcinoma) because of its relatively favourable prognosis, usually without life threatening complications. Similarly, patients with a premalignant condition (e.g. colon polyps) were also excluded.

### Ethical approval

The Medical Ethics Review Committee Erasmus Medical Center Rotterdam (decision number MEC-2018-1172) approved this study. No additional patient consent was required.

### Data collection

Patient and ICU characteristics were collected using the EHR. CR was defined as no detectable malignancy based on data from the EHR. As the 5-year survival of cancer patients is commonly used to describe the prognosis of a malignancy [18,19], we divided patients in CR into two groups: CR < 5 year and CR > 5 year. A metastatic solid malignancy was defined as a solid tumour with the presence of cancer cells in non-primary organ(s) or in distant lymph nodes, as extracted from the EHR.

The comorbidity of patients was measured using the Charlson Comorbidity Index (CCI) [20]. The Eastern Cooperative Oncology Group (ECOG) Performance Status was used to assess performance status in the month to fourteen days before the ICU admission [21]. To evaluate the severity of the acute illness, the Sequential Organ Failure Assessment (SOFA) score was used [22–24]. Unplanned ICU admission was defined as an admission due to an acute medical condition or an admission following emergency surgery or scheduled surgery with intra-operative complications requiring ICU admission. Readmissions were defined as a new ICU admission within 30 days after ICU discharge during the same hospitalization period.

Comfort care was defined as the withdrawal of life-sustaining ICU treatment combined with the initiation of palliative medications when indicated. Short-term mortality was defined as ICU and hospital mortality. Long-term mortality was defined as mortality 2 years after ICU admission.

### Outcomes

The primary objective of the study was to assess long-term mortality and performance status of critically ill cancer patients. Furthermore, to compare the mortality of these patients to patients without active cancer admitted to the ICU unplanned. We used data from the EHR (e.g. medical records by specialists) to determine mortality and performance status of cancer patients 2 years after ICU admission and 2-year mortality in patients without active cancer.

Secondary objectives were ICU and hospital mortality, mortality at 6 months and 1 year following the index ICU admission and the identification of factors associated with 2-year mortality in cancer patients. Further, to assess short-term and long-term mortality of patients in CR. Similarly to the primary outcomes, the EHR was used to determine secondary objectives.

### Statistical analyses

Descriptive statistics were used to describe patient characteristics, ICU characteristics and mortality in the study population. Categorical variables are reported as numbers with percentage. Continuous variables are reported as mean and standard deviation for normally distributed data or, in case of a skewed distribution, median with 25th–75th interquartile range (IQR).

Mortality is reported as numbers with percentage. Performance status is reported as mean and standard deviation for normally distributed data or, in case of a skewed distribution, median with 25th–75th interquartile range. To compare the 2-year mortality and relevant patient and ICU characteristics (i.e. age, gender, SOFA score), we used Pearson’s Chi-square tests or the Fisher’s exact tests for categorical variables and Independent Samples T-Tests (normal distribution) or the Mann-Whitney U tests (skewed distributions) for continuous variables. A statistical test with a two tailed p value ≤ 0.05 was considered as significant.

Univariable binary logistic regression was used to assess the association between long-term mortality and the following variables: age, gender, ECOG PS before ICU admission, status of the malignancy (i.e. active, CR< 5 year, CR>5 year), malignancy type (i.e. solid or hematological), admission reason, metastatic solid disease, stem cell transplantation, SOFA score, sepsis and cancer treatment during ICU admission. Subsequently, variables with a p-value <0.2 in the univariable analysis were evaluated in a multivariable binary logistic regression analysis, except for variables with a small sample size or in case of collinearity. Data were analysed by using IBM®SPSS® Statistics 24.0 (IBM, Chicago, IL, USA).

## Results

A total of 2486 patients were admitted to the ICU in 2017, of whom 1046 (42%) were unplanned admissions. Of the latter, 125 patients (12%) were diagnosed as cancer patients. Patient characteristics are shown in Table 1.

**Table 1:** Patient and ICU characteristics study population.

	Study population (n = 125)
**Patient Characteristics**	
Age	66 [59–73]
Male	84 (67.2%)
*Comorbidity*	
Patients with comorbidity	116 (92.8%)
CCI^a^	3 [2–6]
Age-adjusted CCI	6 [4–8]
ECOG performance status^b^	1 [1–3]
*Type malignancy*	
Solid malignancy	101 (80.2%)
Hematological malignancy	21 (16.8%)
Both solid and hematological malignancy	1 (0.8%)
Unknown type	2 (1.6%)
**Characteristics ICU admission**	
*Admission reason*	
Emergency surgery	22 (17.6%)
Medical	100 (80%)
Both	3 (2.4%)
*Medical admission reasons*	
Respiratory insufficiency	29 (23.2%)
Sepsis	15 (12%)
Post cardiopulmonary resuscitation	5 (4%)
Neurological (non-traumatic)	10 (8%)
Other	24 (19.2%)
Combination	20 (16%)
Readmissions	32 (25.6%)
SOFA^c^ score	7 [5–10]
Mechanical ventilation	83 (66.4%)
Vasopressors	90 (72%)
Renal Replacement Therapy	27 (21.6%)
Sepsis during ICU admission	54 (43.2%)
Cancer treatment during ICU admission	8 (6.4%)
Length of ICU stay (days)	3 [1–11]

* Categorical variables: Mean (%). Continuous variables: Mean (standard deviation) for normally distributed data, skewed distribution, median [25th–75th IQR].

a. CCI; Carlson Comorbidity Index (CCI).

b. ECOG PS: ECOG: Eastern Cooperative Oncology Group (ECOG) performance status.

c. SOFA; Sequential Organ Failure Assessment score (SOFA score).

The majority of the cancer patients had a diagnoses of a solid malignancy (101, 80.2%). The types of malignancies are shown in S1 Table. The main reason for ICU admission was medical (80%), where 23% of them were admitted for respiratory insufficiency (Table 1).

The long-term mortality in cancer patients was 72% (Table 2). The median ECOG performance status in survivors with a known performance status was 1 (IQR 0–1, Table 2), 77% of the survivors with a known ECOG performance status had an ECOG performance status of 0 or 1. In 10.4%, the ECOG performance status was unknown.

**Table 2:** Outcome study population.

	Study population (n = 125)
**Mortality**	
ICU	40 (32%)
Hospital	55 (44%)
6 months	75 (60%)
1-year	84 (67.2%)
2-year	90 (72%)
Unknown	0 (0%)
**ECOG performance status**^**a**^	
Post IC	3 [3–-4]
Post hospital	2 [2–3]
6 months post ICU	1 [1–2]
1-year post ICU	1 [0–2]
2-year post ICU	1 [0–1]
**ECOG performance status 2-years post ICU**	
0	7 (5.6%%)
1	10 (8%)
2	2 (1.6%)
3	2 (1.6%)
4	1 (0.8%)
5	90 (72%)
Unknown	13 (10.4%)

* Categorical variables: Mean (%). Continuous variables: Mean (standard deviation) for normally distributed data, skewed distribution, median [25th–75th IQR].

a. ECOG PS: ECOG: Eastern Cooperative Oncology Group (ECOG) performance status.

Cancer patients were significantly older compared to non-cancer patients, while gender and SOFA score were similar (Table 3). Except for ICU mortality, mortality was significantly higher in cancer patients compared to non-cancer patients at every time point analysed (Table 3). Having an active malignancy was independently associated with long-term mortality (S2 and S3 Tables). During ICU stay, comfort care was more frequently initiated in cancer patients than in non-cancer patients (32.8% versus 22.1% respectively, p <0.001).

**Table 3:** Characteristics and outcome of study population compared to population without malignancy.

	Study population (n = 125)	Without malignancy (n = 921)	P-value
Age	66 [59–73]	58 [46–68]	<0.001*
Male	84 (67.2%)	577 (62.9%)	0.37
SOFA score^a^ at ICU admission	7 [5–10]	8 [5–10]	0.79
SOFA score^a^ at ICU death	12 [8–16]	10 [8–16]	0.70
ICU mortality	40 (32%)	223 (24.2%)	0.06
Hospital mortality	55 (44%)	286 (31.1%)	0.004*
6 months mortality	75 (60%)	341 (37%)	<0.001*
1-year mortality	84 (67.2%)	363 (39.4%)	<0.001*
2-year mortality	90 (72%)	391 (42.5%)	<0.001*
Unknown	0 (0%)	32 (3.5%)	
Start comfort care ICU	41 (32.8%)	204 (22.1%)	<0.001*

* Categorical variables: Mean (%). Continuous variables: Mean (standard deviation) for normally distributed data, skewed distribution, median [25th–75th IQR].

* P- value; probability value, a p-value of < 0.05 was considered statistically significant, marked by an Asterisk.

a. SOFA; Sequential Organ Failure Assessment score (SOFA score).

The binary univariable logistic regression analysis of the study population yielded a p <0.2 for the following variables: age, ECOG PS before ICU admission, readmission, SOFA score at admission and cancer treatment during ICU admission (Table 4).

**Table 4:** Univariable binary logistic regression analysis study population 2-year mortality.

	Patient cases	Mortality	OR^a^	95% CI^b^	P-value^c^
Age	-	-	1.03	0.99–1.06	0.12
Gender (male)	84 (67.2%)	63 (75%)	1.56	0.69–3.51	0.29
Comorbidity (CCI)^d^	-	-	0.90	0.76–1.07	0.23
ECOG PS^e^ before ICU					
0 (ref)	29 (23.2%)	17 (58.6.8%)	-	-	-
1	35 (28%)	25 (71.4%)	1.77	1.17–6.04	0.29
2	23 (18.4%)	19 (82.6%)	3.35	1.55–10.80	0.07
3	25 (20%)	19 (76%)	2.24	1.24–6.71	0.18
4	6 (4.8%)	6 (100%)	-	-	-
Solid malignancy (ref)	101 (80.8%)	72 (71.3%)			
Hematological malignancy	21 (16.8%)	15 (71.4%)	1.01	0.36–2.85	0.99
Emergency surgery (ref)	22 (17.6%)	16 (72.7%)			
Medical reasons	100 (80%)	71 (71%)	0.92	0.33–2.58	0.87
Metastatic malignancy	54 (43.2%)	40 (74.1%)	1.30	0.55–3.08	0.55
Stem cell transplantation	4 (3.2%)	3 (75%)	1.17	0.12–11.67	0.89
Readmissions	32 (25.6%)	19 (59.4%)	0.45	0.19–1.06	0.07
SOFA score ^f^	-	-	1.13	0.99–1.29	0.06
Sepsis	54 (43.2%)	41 (75.9%)	1.42	0.64–3.16	0.40
Cancer treatment during ICU	8 (6.4%)	4 (50%)	0.36	0.09–1.53	0.17

a OR; Odds ratio.

b CI; confidence interval.

c P- value; probability value, a p-value of < 0.05 was considered statistically significant, marked by an Asterisk *.

d CCI; Carlson Comorbidity Index (CCI).

e ECOG PS: ECOG: Eastern Cooperative Oncology Group (ECOG) performance status.

f SOFA; Sequential Organ Failure Assessment score (SOFA score).

After adjustment for confounders, only an ECOG performance status before ICU admission of 2 (OR 8.94; 95% CI 1.21–65.89) was associated long-term mortality (Table 5). Readmissions (OR 0.16 95% CI 0.04–0.60) and cancer treatment during ICU (OR 0.04 95% CI 0.004–0.53) were associated with 2-year survival.

**Table 5:** Multivariable binary logistic regression analysis study population 2-year mortality.

	OR^a^	95% CI^b^	P-value^c^
Age	1.03	0.98–1.09	0.27
Gender (male)	1.28	0.39–4.21	0.68
CCI^d^	0.83	0.62–1.10	0.19
ECOG PS^e^ 0 (ref)			
1	2.23	0.53–9.36	0.28
2	8.94	1.21–65.89	0.03*
3	3.19	0.81–19.01	0.09
Readmissions	0.16	0.04–0.60	0.006*
SOFA^f^ score	1.11	0.95–1.31	0.20
Sepsis	1.65	0.51–5.26	0.40
Cancer treatment during ICU	0.04	0.004–0.53	0.01*

a OR; Odds ratio.

b CI; confidence interval.

c P- value; probability value, a p-value of < 0.05 was considered statistically significant, marked by an Asterisk *.

d CCI; Carlson Comorbidity Index (CCI).

e ECOG PS: ECOG: Eastern Cooperative Oncology Group (ECOG) performance status.

f SOFA; Sequential Organ Failure Assessment score (SOFA score).

Factors independently associated hospital mortality were ECOG performance status before ICU admission (1: OR 8.60 95% CI 1.97–37.60; 2: OR 5.63; 95% CI 1.09–29.11; 3: OR 14.22; 3.02–66.90 and 4: 20.11 95% CI 1.63–248.8) and SOFA score (OR 1.21; 1.04–1.40) (S4 and S5 Tables).

No differences in mortality and ECOG performance status after ICU admission between patients with an active malignancy and patients in CR were seen (S6 Table). While long-term mortality was significantly higher in patients in CR than in patients without a malignancy, no statistical difference in short-term mortality was seen (S7 Table).

## Discussion

Clinicians may have doubts about the appropriateness of an ICU admissions in acutely ill cancer patients. Reliable literature about long-term mortality and morbidity of cancer patients admitted to the ICU unplanned is essential in order to manage outcome expectations of health care providers, patients and relatives. In our study we found that in a population of cancer patients acutely admitted to the ICU, a poor functional status, as measured by the ECOG performance status, and the severity of illness on admission, as measured by the SOFA score, to be independently associated with hospital mortality. Only performance status was independently associated with long-term mortality in these patients. In addition, our study results suggest that having an active malignancy influences long-term mortality as well.

Approximately three-quarter of the cancer patients die within 2 years after ICU admission. The long-term mortality is significantly higher than the long-term mortality of patients without active cancer. One explanation for this difference could be the weakened condition with a poor ECOG performance status directly after ICU discharge in cancer patients. The performance status was still reduced at hospital discharge, probably influencing long-term mortality. Earlier literature with older data suggested the influence of having a malignancy on long-term mortality [25,26]. Despite innovative diagnostics and treatments, our study shows that having a malignancy still influences long-term mortality.

The finding that performance status (measured by the ECOG performance status) was independently associated with short-term mortality and long-term mortality [27–31] is in line with other literature. Similar to our study, severity of illness (measured by the SOFA score) has been described in literature as predictor for short-term mortality in patients with a malignancy [26,32,33]. However, literature suggested an association between long-term mortality and SOFA score as well [28,29,34]. Most of these studies have a maximal follow up of 1 year after ICU admission. Our study shows that SOFA score is important for short-term mortality, but loses its predicting value for long-term mortality in ICU patients with cancer. To a lesser degree, age was associated with mortality in our study. After an ICU admission, aging is associated with an increased risk of mortality in the 3 years after hospital discharge in other literature as well [35].

The decision to deny a cancer patient ICU admission should be based on the performance status before admission and the severity of illness at time of ICU admission. In addition, ICU admission should also depend on the prognostic expectations of the malignancy. Not only ICU physicians, but also hematologists and oncologists should discuss this with patients and relatives before an ICU admission. However, prognostication at individual patient level by clinicians remains difficult [1,36]. It is especially difficult in cancer patients, due to the many factors related to the underlying malignancy (e.g. stage, type, hormone receptor status), and the estimation whether the patient will be able to receive future anti-cancer treatment after ICU admission [1,36]. Moreover, poor communication regarding outcome and expectations towards other health care providers or the patient and relatives has been described, either due to insufficient knowledge concerning prognostication or communication, or due to difficulty with sharing a poor prognosis [36–38]. To improve prognostication and communication, good collaboration with open communication in multidisciplinary meetings and joint education regarding expectations and outcomes is essential [36].

The hospital mortality in our study population was similar to other European literature [2,4,5,39–42]. In contrast, the long-term mortality in our study was higher than in other studies [25–27,31,32,36,43]. An explanation may be the difference in case-mix. We included only unplanned ICU admissions, while most other studies included patients with planned ICU admissions as well. In addition, our study population had often a higher SOFA score and received more often organ support.

We found that during ICU admission, comfort care was more often started in cancer patients than in patients without cancer. In our study, comfort care was more frequently initiated than in the study population of a specialised Portuguese Cancer institute (13%) [42]. Besides differences in case-mix, end-of-life (EoL) decisions could be influenced by many factors, such as religious beliefs, cultural backgrounds, and the ethical climate on the ICU [44]. Therefore, the presence of malignancy could also influence EoL decisions by clinicians. By starting comfort care, we might spare patients, who will die regardless of ICU treatment, from invasive treatments, such as the insertion of multiple intravenous catheters or prolonged mechanical ventilation. Another explanation exists. Literature shows that prognostication for an individual patient remains difficult [36], and suggests the existence of self-fulfilling prophecy (SFP) in medical decision making, especially in EoL decisions [45]. As starting comfort care (the withdrawal of life-sustaining ICU treatment combined with the initiation of palliative medications when indicated) inevitable leads to death, we might deprive cancer patients the possibility of prolonged survival if we misjudge the prognosis of an individual patient. To prevent such a misjudgement, EoL decisions should be made in a multidisciplinary meeting.

We did not find a statistically significant difference in either short- or long-term mortality between patients with an active malignancy and patients in CR. However, a clinically relevant difference in 1-year mortality (67.2% vs 58.2%) and 2-year mortality (72% vs 59.5%) was seen between these groups. This finding suggests that for short-term mortality, other factors such as comorbidity and severity of illness should be considered as important factor for outcome, while the status of the malignancy plays an increasingly important role in long-term mortality. Patients in CR showed higher long-term mortality rates when compared to the patients without cancer. This finding may suggest an influence of having a malignancy in CR, and therefore previous cancer treatment, on long-term mortality. To our knowledge, the mortality of patients with an active malignancy compared to those in CR and the general population has not been directly described in the current literature.

Despite the high mortality, we think it is important to note that in our study population, the majority of the survivors had a good performance status 2 years after ICU admission. Seventy-seven percent of the patients with a known ECOG performance status scored 0 or 1. Our findings are consistent with Zafra and co-workers; in their study 79% of the survivors at 1 year after ICU admission showed an ECOG performance status of 0–2 [46].

### Limitations

First, the generalizability of our findings is limited due to the relatively small sample size of our study and the heterogeneity of the study population. Given the small sample size, dividing our study population into smaller groups would compromise the quality of the statistical analyses, and thus, the quality of research. Future studies with a large sample size should examine outcome of patients per cancer type. Literature shows clearly late negative effects of cancer therapies, even decades after completion of the cancer treatment [13–17]. We therefore did not exclude patients with a malignancy in their distant past, which caused a wide variation of duration of the CR.

Third, selection bias might have influenced our outcome, as our ICU physicians already made an admission decision before ICU admission. Nevertheless, our study population accounted for 12% of the unplanned ICU admissions, which is comparable to other studies.

Fourth, although performance status is not equal to quality of life, the scarcity of studies with long outcome data make our data important.

Fifth, this is a single center study, that limits external validity of the data, even though our institute is the largest university hospital in the Netherlands, covering oncologic care for about 3 million people.

Last, given this was a retrospective study, all limitations of these kind of studies apply.

## Conclusion

In our study, the majority of the survivors have a good performance status 2 years after ICU admission. However, at that point, three-quarter of these cancer patients had died, and mortality in cancer patients was significantly higher than in patients without cancer. ICU admission decisions in acutely ill cancer patients should be based on performance status, severity of illness and long-term prognosis, and should be communicated in the shared decision making. An ICU admission decision should not solely be based on the presence of a malignancy.

## Supporting information

S1 Checklist STROBE checklist. (DOCX)

S1 Table Numbers and percentage malignancy types. (DOC)

S2 Table Univariable binary logistic regression analysis total ICU population: 2-year mortality. (DOC)

S3 Table Multivariable binary logistic regression analysis total ICU population: 2-year mortality. (DOC)

S4 Table Univariable binary logistic regression analysis study population: Hospital mortality. (DOC)

S5 Table Multivariable binary logistic regression analysis study population: Hospital mortality. (DOC)

S6 Table Outcome patients with an active malignancy compared to outcome patients in complete remission. (DOC)

S7 Table Outcome patients in complete remission compared to outcome patients without a malignancy. (DOC)

S8 Table Univariable binary logistic regression analysis study population: 1-year mortality. (DOC)

S9 Table Multivariable binary logistic regression analysis study population: 1-year mortality. (DOC)

S10 Table Outcome of patients with an active solid malignancy versus patients with an active hematological malignancy. (DOC)

S1 Dataset (CSV)

S2 Dataset (CSV)

S3 Dataset (CSV)

## Abbreviations

ICU: Intensive Care Unit
CR: Complete remission
IQR: Interquartile range
SOFA score: Sequential Organ Failure Assessment Score
ECOG performance status or ECOG PS: Eastern Cooperative Oncology Group Performance Status
CCI: Charlson Comorbidity Index
OR: Odds ratio
CI: Confidence interval
EoL: End-of-Life
SFP: Self-fulfilling prophecy
