# Supplementary material for: Assessment of mortality and performance status in critically ill cancer patients: A retrospective cohort study
Source: PLoS One. 2021 Jun 11;16(6):e0252771. doi: 10.1371/journal.pone.0252771 (PMC8195393; doi:10.1371/journal.pone.0252771)
Supplement: S1 Table — (DOC) [file pone.0252771.s002.doc]

**S1. Supplementary material Table 1: Numbers and percentage Malignancy types**

| **Type malignancy** | **Active malignancy**  **(n=125)** |  | **CR^a^ < 5 year**  **(n=41)** | **CR^a^ > 5 year**  **(n = 33)** |
| --- | --- | --- | --- | --- |
| **Solid malignancy**  Colorectal carcinoma  Esophageal carcinoma  Lung carcinoma  Bladder carcinoma  Prostate carcinoma  Breast carcinoma  Cholangiocarcinoma  Larynx carcinoma  Gastric carcinoma  Malignancy of central nervous system  Melanoma  Neuroendocrine tumor  Hepatocellular carcinoma  Pancreatic carcinoma  Mesothelioma  Sarcoma  Other  Unknown | 17 (13.6%)  14 (11.2%)  12 (9.6%)  4 (3.2%)  5 (4.0%)  0 (0%)  10 (8.0%)  1 (0.8%)  3 (2.4%)  4 (3.2%)  3 (2.4%)  4 (3.2%)  3 (2.4%)  0 (0%)  3 (2.4%)  1 (0.8%)  20 (16.0%)  2 (1.6%) |  | 3 (7.3%)  7 (17.1%)  3 (7.3%)  2 (4.9%)  3 (7.3%)  4 (9.8%)  0 (0%)  1 (2.4%)  2 (4.9%)  0 (0%)  0 (0%)  0 (0%)  1 (2.4%)  4 (9.8%)  0 (0%)  0 (0%)  6 (14.6%)  0 (0%) | 2 (6.1%)  1 (3.0%)  1 (3.0%)  7 (21.2%)  4 (12.1%)  5 (15.2%)  0 (0%)  3 (9.1%)  0 (0%)  0 (0%)  1 (3.0%)  0 (0%)  0 (0%)  0 (0%)  0 (0%)  1 (3.0%)  9 (27.3%)  0 (0%) |
| **Hematological malignancy**  Acute myeloid leukemia  Non-Hodgkin Lymphoma  Hodgkin Lymphoma  Acute lymphocytic leukemia  Other | 6 (4.8%)  2 (1.6%)  0 (0%)  3 (2.4%)  11 (8.8%) |  | 1 (2.4%)  2 (4.9%)  3 (7.3%)  2 (4.9%)  3 (7.3%) | 0 (0%)  0 (0%)  2 (6.1%)  2 (6.1%)  1 (3.0%) |

1. CR: complete remission
2. Note; some patients have more than 1 malignancy
