# Supplementary material for: Assessment of mortality and performance status in critically ill cancer patients: A retrospective cohort study
Source: PLoS One. 2021 Jun 11;16(6):e0252771. doi: 10.1371/journal.pone.0252771 (PMC8195393; doi:10.1371/journal.pone.0252771)
Supplement: S2 Table — (DOC) [file pone.0252771.s003.doc]

**S2. Supplementary material Table 2: Univariable binary logistic regression analysis total ICU population: 2-year mortality**

|  | **Patient cases** | **Mortality** | **OR ^a^** | **95% CI ^b^** | **P-value ^c^** |
| --- | --- | --- | --- | --- | --- |
| Age | - | - | 1.04 | 1.03-1.05 | < 0.001* |
| Gender (male) | 630 (60.2%) | 307 (48.7%) | 1.15 | 0.90-1.48 | 0.29 |
| SOFA score ^d^ | - | - | 1.12 | 1.07-1.18 | < 0.001* |
| No malignancy (ref)  Active malignancy  Complete remission < 5yr  Complete remission > 5yr | 793 (75.8%)  125 (12%)  41 (3.9%)  33 (3.2%) | 332 (41.9%)  90 (72%)  27 (65.9%)  19 (57.6%) | 3.57  2.68  1.88 | 2.36-5.41  1.38-5.19  0.93-3.81 | <0.001*  0.003*  0.08 |

a OR; Odds ratio

b CI; confidence interval

c P- value; probability value, a p-value of < 0.05 was considered statistically significant, marked by an Asterisk *

d SOFA; Sequential Organ Failure Assessment score (SOFA score)
