# Supplementary material for: Assessment of mortality and performance status in critically ill cancer patients: A retrospective cohort study
Source: PLoS One. 2021 Jun 11;16(6):e0252771. doi: 10.1371/journal.pone.0252771 (PMC8195393; doi:10.1371/journal.pone.0252771)
Supplement: S3 Table — (DOC) [file pone.0252771.s004.doc]

**S3. Supplementary material Table 3: Multivariable binary logistic regression analysis total ICU population: 2-year mortality**

|  | **OR ^a^** | **95% CI ^b^** | **P-value ^c^** |
| --- | --- | --- | --- |
| Age | 1.04 | 1.02-1.05 | <0.001* |
| SOFA score ^d^ | 1.15 | 1.09-1.22 | <0.001* |
| No malignancy (ref)  Active malignancy  Complete remission < 5yr  Complete remission > 5yr | 3.75  3.75  2.11 | 2.21-6.34  1.62-8.65  0.92-4.84 | <0.001*  0.002*  0.08 |

a OR; Odds ratio

b CI; confidence interval

c P- value; probability value, a p-value of < 0.05 was considered statistically significant, marked by an Asterisk *

d SOFA; Sequential Organ Failure Assessment score (SOFA score)
