# Supplementary material for: Assessment of mortality and performance status in critically ill cancer patients: A retrospective cohort study
Source: PLoS One. 2021 Jun 11;16(6):e0252771. doi: 10.1371/journal.pone.0252771 (PMC8195393; doi:10.1371/journal.pone.0252771)
Supplement: S4 Table — (DOC) [file pone.0252771.s005.doc]

**S4. Supplementary material Table 4: Univariable binary logistic regression analysis study population: hospital mortality**

|  | **Patient cases** | **Mortality** | **OR ^a^** | **95% CI ^b^** | **P-value ^c^** |
| --- | --- | --- | --- | --- | --- |
| Age | - | - | 1.03 | 0.99-1.06 | 0.11 |
| Gender (male) | 84 (67.2%) | 41 (48.8%) | 2.05 | 0.94-4.50 | 0.07 |
| Comorbidity (CCI) ^d^ | - | - | 0.81 | 0.69-0.96 | 0.01* |
| ECOG ^e^ PS before ICU  0 (ref)  1  2  3  4 | 29 (23.2%)  35 (28%)  23 (18.4%)  25 (20%)  6 (4.8%) | 7 (24.1%)  14 (40%)  8 (34.8%)  17 (68%)  5 (83.3%) | -  2.10  1.68  6.68  15.7 | -  0.71-6.21  0.50-5.61  2.02-22.08  1.56-158.2 | -  0.18  0.40  0.002*  0.02* |
| Solid malignancy (ref)  Hematological malignancy | 101 (80.8%)  21 (16.8%) | 40 (39.6%)  13 (61.9%) | 2.48 | 0.94-6.52 | 0.07 |
| Emergency surgery (ref)  Medical reasons | 22 (17.6%)  100 (80%) | 7 (31.8%)  46 (46%) | 1.83 | 0.69-4.86 | 0.23 |
| Metastatic malignancy | 54 (43.2%) | 23 (42.6%) | 1.35 | 0.61-3.01 | 0.46 |
| Stem cell transplantation | 4 (3.2%) | 2 (50%) | 1.33 | 0.18-9.73 | 0.78 |
| Readmissions | 32 (25.6%) | 13 (40.6%) | 0.87 | 0.38-1.96 | 0.73 |
| SOFA score ^f^ | - | - | 1.13 | 1.01-1.26 | 0.03* |
| Sepsis | 54 (43.2%) | 28 (51.9%) | 1.86 | 0.91-3.83 | 0.09 |
| Cancer treatment during ICU | 8 (6.4%) | 2 (25%) | 0.42 | 0.08-2.15 | 0.30 |

a OR; Odds ratio

b CI; confidence interval

c P- value; probability value, a p-value of < 0.05 was considered statistically significant, marked by an Asterisk *

d CCI; Carlson Comorbidity Index (CCI)

e ECOG PS: ECOG: Eastern Cooperative Oncology Group (ECOG) performance status

f SOFA; Sequential Organ Failure Assessment score (SOFA score)
