# Supplementary material for: Assessment of mortality and performance status in critically ill cancer patients: A retrospective cohort study
Source: PLoS One. 2021 Jun 11;16(6):e0252771. doi: 10.1371/journal.pone.0252771 (PMC8195393; doi:10.1371/journal.pone.0252771)
Supplement: S5 Table — (DOC) [file pone.0252771.s006.doc]

**S5. Supplementary material Table 5: Multivariable binary logistic regression analysis study population: hospital mortality**

|  | **OR ^a^** | **95% CI ^b^** | **P-value ^c^** |
| --- | --- | --- | --- |
| Age | 1.05 | 0.99-1.10 | 0.06 |
| Gender (male) | 0.48 | 0.17-1.36 | 0.17 |
| CCI ^d^ | 0.67 | 0.52-0.87 | 0.003* |
| ECOG ^e^ PS 0 (ref)  1  2  3  4 | 8.60  5.63  14.22  20.11 | 1.97-37.60  1.09-29.11  3.02-66.90  1.63-248.82 | 0.004*  0.04*  0.001*  0.02* |
| Hematological malignancy | 1.23 | 0.52-2.91 | 0.64 |
| SOFA score ^f^ | 1.21 | 1.04-1.40 | 0.01* |
| Sepsis | 1.05 | 0.36-3.04 | 0.93 |

a OR; Odds ratio

b CI; confidence interval

c P- value; probability value, a p-value of < 0.05 was considered statistically significant, marked by an Asterisk *

d CCI; Carlson Comorbidity Index (CCI)

e ECOG PS: ECOG performance status: Eastern Cooperative Oncology Group (ECOG) performance status before ICU

f SOFA; Sequential Organ Failure Assessment score (SOFA score)
