# Supplementary material for: Assessment of mortality and performance status in critically ill cancer patients: A retrospective cohort study
Source: PLoS One. 2021 Jun 11;16(6):e0252771. doi: 10.1371/journal.pone.0252771 (PMC8195393; doi:10.1371/journal.pone.0252771)
Supplement: S6 Table — (DOC) [file pone.0252771.s007.doc]

**S6. Supplementary material Table 6: Outcome patients with an active malignancy compared to outcome patients in complete remission**

|  | **Active malignancy**  **(n=125)** | **Complete remission (n=79)** | **p-value** |
| --- | --- | --- | --- |
| **Mortality** |  |  |  |
| ICU | 40 (32%) | 21 (26.6%) | 0.41 |
| Hospital | 55 (44%) | 34 (43%) | 0.89 |
| 6 months | 75 (60%) | 42 (53.2%) | 0.34 |
| 1-year | 84 (67.2%) | 46 (58.2%) | 0.19 |
| 2-year | 90 (72%) | 47 (59.5%) | 0.06 |
| **ECOG performance status** |  |  |  |
| Post IC | 3 [3-4] | 3 [3-4] | 0.69 |
| Post hospital | 2 [2-3] | 2 [2-3] | 0.29 |
| 6 months | 1 [1-2] | 2 [1-2] | 0.18 |
| 1-year | 1 [0-2] | 1 [1-2] | 0.11 |
| 2-years | 1 [0-1] | 1 [1-2] | 0.47 |

*5 patients had CR < 5 year and CR > 5 year
