# Supplementary material for: Assessment of mortality and performance status in critically ill cancer patients: A retrospective cohort study
Source: PLoS One. 2021 Jun 11;16(6):e0252771. doi: 10.1371/journal.pone.0252771 (PMC8195393; doi:10.1371/journal.pone.0252771)
Supplement: S7 Table — (DOC) [file pone.0252771.s008.doc]

**S7. Supplementary material Table 7: Outcome patients in complete remission compared to outcome patients without a malignancy**

|  | **Complete remission**  **(n=79)** | **Without malignancy**  **(n = 825)** | **p-value^a^** |
| --- | --- | --- | --- |
| **Mortality** |  |  |  |
| ICU | 22 (27.8%) | 196 (23.8%) | 0.42 |
| Hospital | 35 (44.3%) | 244 (29.6%) | 0.07 |
| 6 months | 43 (54.4%) | 288 (34.9%) | 0.001* |
| 1-year | 47 (59.5%) | 305 (37%) | < 0.001* |
| 2-year | 48 (60.8%) | 332 (40.2%) | 0.001* |

a P- value; probability value, a p-value of < 0.05 was considered statistically significant, marked by an Asterisk *
