# Supplementary material for: Assessment of mortality and performance status in critically ill cancer patients: A retrospective cohort study
Source: PLoS One. 2021 Jun 11;16(6):e0252771. doi: 10.1371/journal.pone.0252771 (PMC8195393; doi:10.1371/journal.pone.0252771)
Supplement: S8 Table — (DOC) [file pone.0252771.s009.doc]

**S8. Supplementary material Table 8: Univariable binary logistic regression analysis study population: 1-year mortality**

|  | **Patient cases** | **Mortality** | **OR ^a^** | **95% CI ^b^** | **P-value ^c^** |
| --- | --- | --- | --- | --- | --- |
| Age | - | - | 1.03 | 0.99-1.06 | 0.10 |
| Gender (male) | 84 (67.2%) | 60 (71.4%) | 1.77 | 0.81-3.87 | 0.15 |
| Comorbidity (CCI) ^d^ | - | - | 0.91 | 0.77-1.07 | 0.25 |
| ECOG ^e^ PS before ICU  0 (ref)  1  2  3  4 | 29 (23.2%)  35 (28%)  23 (18.4%)  25 (20%)  6 (4.8%) | 16 (55.2%)  24 (68.6%)  16 (69.6%)  19 (76%)  6 (100%) | -  1.77  1.86  2.57  - | -  0.64-4.93  0.59-5.87  0.80-8.32  - | -  0.27  0.29  0.12  - |
| Solid malignancy (ref)  Hematological malignancy | 101 (80.8%)  21 (16.8%) | 66 (65.3%)  15 (71.4%) | 1.33 | 0.47-3.72 | 0.59 |
| Emergency surgery (ref)  Medical reasons | 22 (17.6%)  100 (80%) | 14 (63.6%)  67 (67%) | 1.16 | 0.44-3.04 | 0.76 |
| Metastatic malignancy | 54 (43.2%) | 39 (72.2%) | 1.86 | 0.81-4.25 | 0.14 |
| Stem cell transplantation | 4 (3.2%) | 3 (75%) | 1.48 | 0.15-14.70 | 0.73 |
| Readmissions | 32 (25.6%) | 18 (56.3%) | 0.53 | 0.23-1.21 | 0.13 |
| SOFA score ^f^ | - | - | 1.13 | 0.99-1.28 | 0.07 |
| Sepsis | 54 (43.2%) | 40 (74.1%) | 1.75 | 0.81-3.80 | 0.15 |
| Cancer treatment during ICU | 8 (6.4%) | 4 (50%) | 0.46 | 0.11-1.95 | 0.29 |

a OR; Odds ratio

b CI; confidence interval

c P- value; probability value, a p-value of < 0.05 was considered statistically significant, marked by an Asterisk *

d CCI; Carlson Comorbidity Index (CCI)

e ECOG PS: ECOG: Eastern Cooperative Oncology Group (ECOG) performance status

f SOFA; Sequential Organ Failure Assessment score (SOFA score)
