# Supplementary material for: Assessment of mortality and performance status in critically ill cancer patients: A retrospective cohort study
Source: PLoS One. 2021 Jun 11;16(6):e0252771. doi: 10.1371/journal.pone.0252771 (PMC8195393; doi:10.1371/journal.pone.0252771)
Supplement: S9 Table — (DOC) [file pone.0252771.s010.doc]

**S9. Supplementary material Table 9: Multivariable binary logistic regression analysis study population: 1-year mortality**

|  | **OR ^a^** | **95% CI ^b^** | **P-value ^c^** |
| --- | --- | --- | --- |
| Age | 1.04 | 0.99-1.10 | 0.11 |
| Gender (male) | 0.87 | 0.31-2.48 | 0.80 |
| CCI ^d^ | 0.83 | 0.64-1.07 | 0.15 |
| ECOG ^e^ PS 0 (ref)  1  2  3 | 2.52  4.83  4.95 | 0.68-9.36  0.94-24.78  1.11-22.10 | 0.17  0.06  0.04* |
| Readmissions | 0.24 | 0.07-0.81 | 0.02* |
| SOFA score ^f^ | 1.14 | 0.98-1.32 | 0.10 |
| Sepsis | 1.30 | 0.44-3.81 | 0.63 |

a OR; Odds ratio

b CI; confidence interval

c P- value; probability value, a p-value of < 0.05 was considered statistically significant, marked by an Asterisk *

d CCI; Carlson Comorbidity Index (CCI)

e ECOG PS: ECOG performance status: Eastern Cooperative Oncology Group (ECOG) performance status before ICU

f SOFA; Sequential Organ Failure Assessment score (SOFA score)
