# Supplementary material for: Assessment of mortality and performance status in critically ill cancer patients: A retrospective cohort study
Source: PLoS One. 2021 Jun 11;16(6):e0252771. doi: 10.1371/journal.pone.0252771 (PMC8195393; doi:10.1371/journal.pone.0252771)
Supplement: S10 Table — (DOC) [file pone.0252771.s011.doc]

**S10. Supplementary material Table 10: Outcome of patients with an active solid malignancy versus patients with an active hematological malignancy**

|  | **Active Solid**  **(n=101)** | **Active hematological (n=21)** | **p-value^a^** |
| --- | --- | --- | --- |
| **Patient and ICU characteristics** |  |  |  |
| Age | 67 [60-73] | 61 [50-67] | 0.05 |
| Male | 67 (66.3%) | 15 (71.4%) | 0.65 |
| CCI^b^ | 3 [2-6] | 3 [2-5] | 0.14 |
| ECOG PS^c^ before ICU | 2 [1-3] | 1 [0-3] | 0.32 |
| SOFA score ^d^ | 7 [5-10] | 9 [7-13] | 0.003* |
| **Mortality** |  |  |  |
| ICU | 29 (28.7%) | 10 (47.6%) | 0.09 |
| Hospital | 41 (40.6%) | 13 (61.9%) | 0.07 |
| 6 months | 58 (57.4%) | 15 (71.4%) | 0.23 |
| 1-year | 66 (65.3%) | 15 (71.4%) | 0.59 |
| 2-year | 72 (71.3%) | 15 (71.4%) | 0.99 |
| **ECOG performance status^c^** |  |  |  |
| Post IC | 3 [3-4] | 3[3-4] | 0.98 |
| Post hospital | 2 [2-3] | 3 [2-3] | 0.72 |
| 6 months | 1 [1-2] | 1 [1-2] | 0.60 |
| 1-year | 1 [1-2] | 1 [1-1] | 0.48 |
| 2-years | 1 [1-2] | 1 [0-1] | 0.57 |

3 patients had both solid as hematological cancer

a P- value; probability value, a p-value of < 0.05 was considered statistically significant, marked by an Asterisk *

b CCI; Carlson Comorbidity Index (CCI)

c ECOG PS: ECOG: Eastern Cooperative Oncology Group (ECOG) performance status

d SOFA; Sequential Organ Failure Assessment score (SOFA score)
